# Challenges in EGFRvIII Detection in Head and Neck Squamous Cell Carcinoma

**DOI:** 10.1371/journal.pone.0117781

**Published:** 2015-02-06

**Authors:** Sarah E. Wheeler, Ann Marie Egloff, Lin Wang, C. David James, Peter S. Hammerman, Jennifer R. Grandis

**Affiliations:** 1 Department of Pathology, University of Pittsburgh, Pittsburgh, PA, United States of America; 2 Department of Otolaryngology, University of Pittsburgh and University of Pittsburgh Cancer Institute, Pittsburgh, PA, United States of America; 3 Department of Neurological Surgery, Northwestern University, Chicago, IL, United States of America; 4 Dana-Farber Cancer Institute, Harvard Medical School, Boston, MA, United States of America; 5 Department of Pharmacology & Chemical Biology, University of Pittsburgh, Pittsburgh, PA, United States of America; Queen Mary University of London, UNITED KINGDOM

## Abstract

**Objective:**

Head and neck squamous cell carcinoma (HNSCC) accounts for more than 5% of all cancers worldwide. The mortality rate of HNSCC has remained unchanged (approximately 50%) over the last few decades. Ubiquitous overexpression of wild type EGFR in many solid tumors has led to the development of EGFR targeted therapies. EGFR can be constitutively activated via several mechanisms including the truncated, EGFR variant III isoform (EGFRvIII). EGFRvIII lacks exons 2–7 and has been reported to be present in up to 20–40% of HNSCC. EGFRvIII has been shown to contribute to cetuximab resistance. The mechanisms leading to EGFRvIII expression in HNSCC are unknown. The present investigation was undertaken to determine the etiology of EGFRvIII in HNSCC.

**Materials and Methods:**

Fixed HNSCC and glioma tissues were analyzed by fluorescence in situ hybridization for EGFR amplification. DNA and RNA from fresh frozen specimens were used to determine the presence of EGFRvIII transcripts and the mechanisms of expression via PCR, RT-PCR and RNA sequencing.

**Results:**

Unlike glioma, EGFRvIII expression in HNSCC did not correlate with EGFR amplification. We found evidence of genomic deletion of the exon 2–7 in 6 of 7 HNSCC cases examined, however, the presence of genomic deletion did not always result in mRNA expression of EGFRvIII. RNA sequencing with automated alignment did not identify EGFRvIII due to microhomology between intron 1 and exon 8. RNA sequencing analyzed by manual alignment methods did not correlate well with RT-PCR and PCR findings.

**Conclusion:**

These findings suggest that genomic deletion as well as additional regulatory mechanisms may contribute to EGFRvIII expression in HNSCC. Further, large scale automated alignment of sequencing are unlikely to identify EGFRvIII and an assay specifically designed to detect EGFRvIII may be necessary to detect this altered form of EGFR in HNSCC tumors.

## Introduction

Head and neck squamous cell carcinoma (HNSCC) accounts for >5% of all cancers worldwide [1] and is among the most common cancers in many developing countries [2]. The mortality rate (~50%) has remained unchanged for decades. Exposure to environmental carcinogens, namely chronic tobacco and alcohol use, are the major risk factors in the development of HNSCC. Infection with the human papillomavirus (HPV) is emerging as a major cause of oropharyngeal cancers, especially in nonsmokers.

Increased understanding of the mechanisms of HNSCC tumorigenesis and progression is important to improving treatment and outcomes. Overexpression of EGFR is found in up to ~90% of HNSCC cases, however, gene amplification occurs in only 10–20% of HNSCC, suggesting alternative mechanisms for increasing HNSCC EGFR expression including transcriptional activation [3,4]. Elevated EGFR expression is associated with oncogenesis and is an independent predictor of poor prognosis in HNSCC [5,6]. The poor prognosis associated with EGFR overexpression prompted the development of EGFR-targeted therapies including the EGFR specific monoclonal antibody cetuximab, which was FDA-approved for HNSCC in 2006, making it the first new HNSCC treatment in 45 years. Despite ubiquitous EGFR expression in HNSCC tumors, only a subset of individuals will respond to cetuximab therapy [7]. The basis for limited cetuximab responses is currently unknown.

EGFR mutations are rare in HNSCC [8]. The most prevalent EGFR alteration reported in HNSCC is the loss of exons 2–7, resulting in the EGFR variant, EGFRvIII [9]. EGFRvIII is unable to bind ligand, signals constitutively and is co-expressed with wild-type (wt) EGFR in several solid tumors [10]. EGFRvIII was first described in glioma where it has been best studied [10]. EGFRvIII signaling plays a role in tumorigenesis and tumor progression [9,11–14] by mediating cell survival, proliferation, motility, invasion and treatment resistance in glioma, breast cancer and HNSCC, among others [15,16].

EGFR gene amplification is present in ~40% of glioblastoma multiforme (GBM) [17], with EGFRvIII almost exclusively expressed in EGFR amplified tumors [10,18]. EGFRvIII has been reported in up to ~40% of HNSCC by IHC and RT-PCR [9,19]. EGFRvIII expression correlates with therapeutic resistance to cetuximab in preclinical HNSCC models as well as a phase II clinical trial [9,12,20]. Increased understanding of the biology of EGFRvIII expression may lead to improved treatment approaches for tumors harboring this alteration. We undertook the present study to determine the mechanism of EGFRvIII expression in HNSCC, with the ultimate goal of optimizing treatment approaches for HNSCC tumors that harbor this EGFR variant.

## Materials and Methods

### EGFRvIII incidence in EGFR amplified tumors

HNSCC patients treated with curative intent for pathologically-confirmed HNSCC were enrolled in an IRB-approved study prior to surgery (n = 154). This cohort, and accompanying tissue microarray (TMA), including the frequency of EGFR gene amplification has been previously described [21]. For the present study, fresh-frozen tissues (25 HNSCC tumors) from the same cohort were evaluated.

### GBM and HNSCC tissues evaluated for DNA and RNA alterations

Patients gave written informed consent to donate tumor tissue. Tissue from GBM cases (n = 6) with EGFR gene amplification and HNSCC cases with and without EGFR gene amplification (n = 31) were collected as fresh frozen tissue and used in sequencing analyses. All tissues were collected under the auspices of a tissue bank protocol approved by the University of Pittsburgh Institutional Review Board and were de-identified. This study was approved by the University of Pittsburgh Institutional Review Board.

### Fluorescence In Situ Hybridization (FISH) and Immunohistochemistry (IHC)

Dual color FISH analysis for EGFR amplification and IHC staining of an EDRN TMA section was previously described [21].

### EGFRvIII PCR detection

Presence of EGFRvIII was detected by RT-PCR as previously described [9]. Confirmatory cDNA sequencing was performed on all resultant EGFRvIII amplified products. The agarose-fractionated amplicon corresponding to the EGFR mutant band was excised and purified according to manufacturer’s instructions using the Qiagen Gel Extraction kit. The DNA product was sequenced BigDye Terminator sequencing premix (Applied Biosystems, Inc.; Carlsbad, CA) with the Applied Biosystems, Inc. 3730xl or 3130xl DNA Analyzer by the DNA Core Facility at the University of Pittsburgh School of Medicine.

### Exon junction sequencing

Total RNA and total DNA were each isolated from fresh-frozen tumors (HNSCC n = 5; GBM n = 6) using the Qiagen Allprep DNA/RNA kit according to manufacturer’s instructions. RNA was reverse transcribed using the SuperScript III Reverse Transcriptase kit with random hexamers (Invitrogen; Carlsbad, CA) according to manufacturer’s instructions with 2.5 ug of RNA input. PCR to characterize exon junctions was performed on cDNA and DNA. GAPDH was used as the control gene for DNA/RNA integrity (see S1 Methods for primer sequences and PCR conditions). The PCR products were separated, excised, purified and sequenced as noted above. Sequencing results were compared with the standard NCBI EGFR sequence NC_000007.13 for DNA and NM_201284 for mRNA via the basic local alignment search tool (Nucleotide BLAST; NCBI).

### Long range PCR

Long range PCR was performed using previously described and validated primers [22,23]. Primers were located every 3 kb in intron 1 of EGFR. DNA and RNA isolation (HNSCC n = 7; GMB n = 4) and total RNA reverse transcription was performed as noted above (see S1 Methods and S1 Table for PCR conditions). The PCR products were separated on 1% agarose gels, excised, purified and sequenced in both directions as noted above. Sequencing results were compared with the standard NCBI EGFR sequence GRCh37.p10 with NT_033968.6 for DNA and NM_005228.3 for mRNA via the basic local alignment search tool (Nucleotide BLAST; NCBI). EGFRvIII alterations were considered present only if the breakpoint was sequenced.

### RNA sequencing

RNA material used to assess RNA alterations above was analyzed for EGFRvIII via RNA seq (n = 26 total samples). RNA from the U87MG vIII high cell line was used as a positive control. mRNA was isolated from total RNA (250 ng total RNA) using the Magnetic poly-A RNA Isolation Module (New England Biolabs) and heat fragmented for 15 minutes at 95 degrees. Fragmented mRNA was used to generate barcoded libraries for mRNA sequencing using the NEB Next Ultra RNA Library Prep Kit for Illumina (New England Biolabs). Libraries were quantified using a High Sensitivity DNA Chip assay (Agilent) and by quantitative PCR for each individual barcode. Libraries were sequenced to a depth of 50 million reads per sample. FASTQ files were aligned to the human genome using PRADA (PMID: 24695405) and resulting BAM files analyzed using the Cufflinks suite (cufflinks.cbcb.umd.edu/). Automated fusion detection was performed using the Broad Institute pipeline (www.broadinstitute.org/cancer/CGA) and manual review of all reads spanning EGFR was undertaken using Integrative Genomics Viewer.

## Results

### EGFRvIII is expressed in both EGFR amplified and unamplified HNSCC

**Fig 1:**
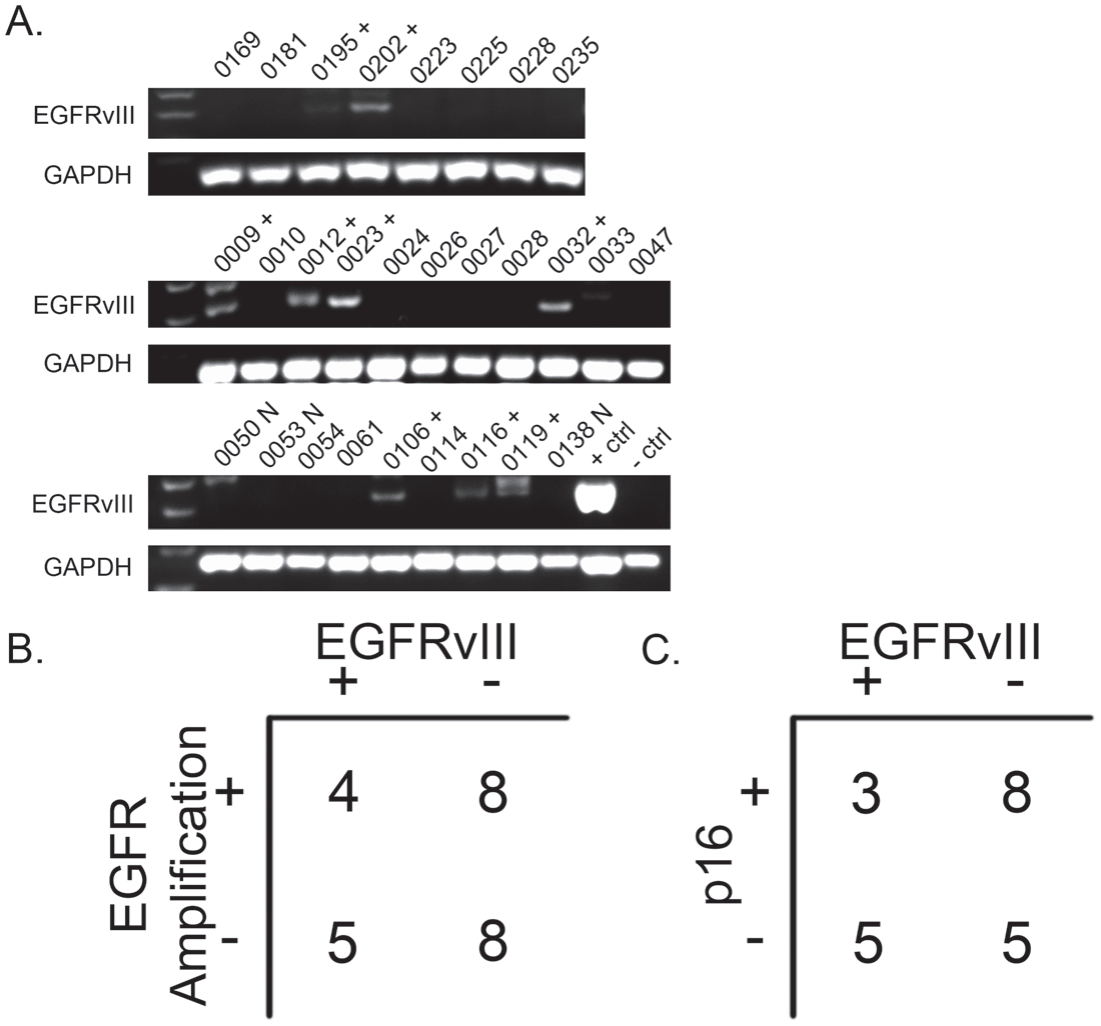
EGFRvIII correlation with EGFR amplification and HPV. A. Twenty five HNSCC tumors with known EGFR gene amplification status (via FISH) were tested for EGFRvIII positivity (28 tumors are shown, three tumors marked with N did not have gene amplification data). RNA was isolated and EGFRvIII and GAPDH were RT-PCR amplified as described in Materials and Methods. 4 of 12 EGFR amplified samples contained EGFRvIII, 5 of 12 of samples without EGFR amplification expressed EGFRvIII. All EGFRvIII bands were excised and sequenced to verify exon 1 to exon 8 joining (samples with confirmed EGFRvIII are denoted by “+”). B. A fisher’s exact test showed a lack of association between EGFRvIII expression and EGFR amplification (p = 0.56). C. A fisher’s exact test showed a lack of association between EGFRvIII expression and p16 expression (p = 0.27).

In glioma, EGFRvIII is present almost exclusively in tumors with EGFR gene amplification [10,18], however a recent study in HNSCC indicated that EGFRvIII expression may not be correlated with EGFR gene amplification [19]. This discordance led us to evaluate a cohort of 25 HNSCC tumors via RT-PCR with (n = 12) or without (n = 13) EGFR amplification to determine if EGFRvIII detection was restricted to HNSCC tumors with EGFR gene amplification. EGFRvIII protein expression was confirmed via IHC in a subset of these tumors as we have previously described [9]. We found that 4/12 tumors with EGFR amplification expressed EGFRvIII compared with 5/13 tumors without EGFR amplification (Fig. 1a, b). These results suggest that EGFR gene amplification is not the only mechanism of EGFRvIII generation in HNSCC.

Expression of p16 has been shown to correlate with HPV positive tumor status in HNSCC [24]. We previously reported p16 status for a clinical HNSCC cohort and here evaluated EGFRvIII expression (as confirmed by RT-PCR and sequencing) and p16 expression. We found that few tumors expressed both p16 and EGFRvIII (3 of 21 tumors, Fig. 1c), which is in agreement with our previous report using an HPV FISH probe [21]. EGFRvIII does not appear to be enriched in HPV positive HNSCC.

### EGFRvIII does not contain splice donor/acceptor mutations in HNSCC or glioma

**Fig 2:**
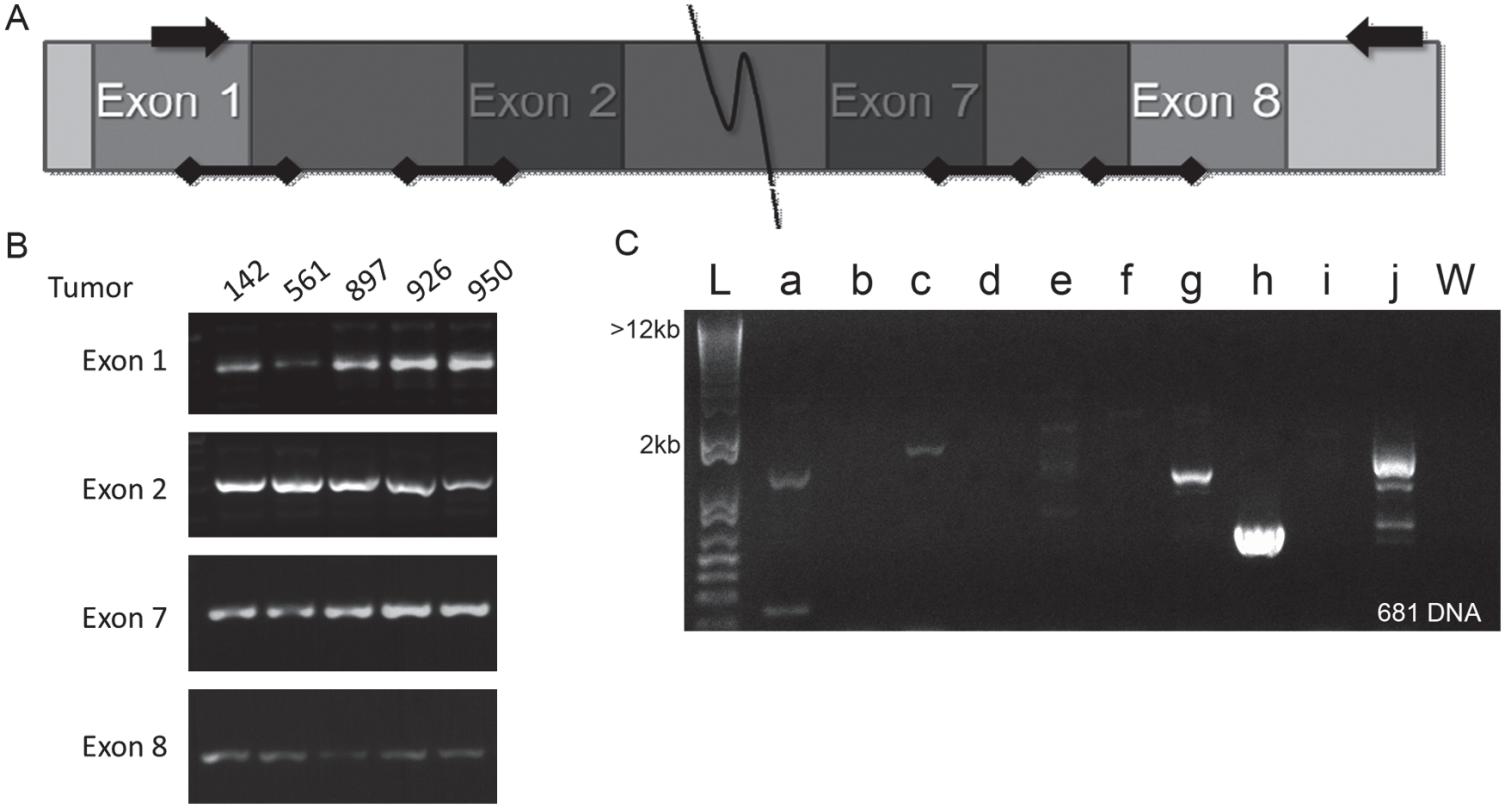
PCR amplification of EGFR for genomic alterations leading to EGFRvIII transcript. A. Schematic of sequencing primers and areas of interest. Arrows indicate the location of the primers used to detect EGFRvIII in genomic DNA and unspliced RNA. Bars with diamond caps indicate areas amplified for splice donor and acceptor mutations. The shaded area is lost in EGFRvIII. B. Representative PCR amplification of the splice donor/acceptor sites of *EGFR* exons 1, 2, 7 and 8 in an EGFRvIII positive HNSCC DNA sample. These bands were excised and sequenced for mutations. C. Representative long-range PCR amplification of *EGFR* intron 1 for a single EGFRvIII positive HNSCC DNA sample. L: base pair marker, W: water control, a-j: primer sets.

EGFRvIII is most frequently detected by mRNA or protein assays and has an in-frame deletion of exons 2–7. We hypothesized that this alteration may be due to alternative splicing, which would be detected at the mRNA and protein levels but not in unspliced RNA or genomic DNA. Point mutations in the splice donor or acceptor sites located in the intron of DNA or RNA-editing of unspliced RNA at the splice donor and acceptor sites could each result in alternative splicing. To determine if there were mutations in these sites we amplified and sequenced the splice donor and acceptor sites in exons 1, 2, 7, and 8 (Fig. 2a) in 4 HNSCC tumors harboring EGFRvIII detected by RT-PCR and 1 HNSCC tumor with EGFRwt only (Fig. 2b). We found no alterations in the genomic DNA or unspliced RNA splice sites indicating that alternative splicing via RNA-editing, or by point mutation of the splice donor and acceptor sites, are unlikely mechanisms of EGFRvIII expression.

Previous IHC studies in our laboratory indicated that EGFRvIII is not as highly expressed in HNSCC as in glioma [9]. We hypothesized that the relatively high expression levels of EGFRwt found in HNSCC may mask alterations in unspliced RNA, which would be more evident in GBM. We assessed 5 fresh frozen glioblastoma samples that expressed EGFRvIII and 1 glioblastoma that expressed only EGFRwt and tested the DNA and unspliced RNA for splice site alterations. No splice site alterations were found in these tumor samples.

### In HNSCC EGFRvIII is not detectable at the DNA or unspliced RNA level

Having determined that EGFRvIII was unlikely to be the consequence of alternative splicing through point mutations, we next considered genomic deletion (despite the lack of significant correlation between EGFRvIII and EGFR gene amplification) or transcriptional dysregulation resulting in an intact gene but an altered pre-mRNA transcript. We explored these possibilities by designing a forward primer located within exon 1 and a reverse primer in the intron following exon 8 (Fig. 2a, primers represented by arrows). We screened DNA and unspliced RNA from representative glioblastoma and HNSCC samples for deletion of exons 2–7. Glioblastomas expressing EGFRvIII at the mRNA level also harbored EGFRvIII in unspliced RNA but not at the genomic level (Table 1). Deletion of exons 2–7 was not detected in the DNA or RNA of a glioblastoma in the absence of EGFRvIII expression. In HNSCC, deletion of exons 2–7 was not detected at the DNA or unspliced RNA level in any of the 5 tumors assessed.

**Table 1:** Presence of Exon 1 to 8 joining in DNA and total RNA.

	HNSCC			GBM	
	Pre mRNA	DNA		Pre mRNA	DNA
HNSCC 1 (-)^a^	ND^b^	ND	GBM 1 (-)	ND	ND
HNSCC 2 (+)	ND	ND	GBM 2 (+)	vIII^c^	ND
HNSCC 3 (+)	ND	ND	GBM 3 (+)	vIII	ND
HNSCC 4 (+)	ND	ND	GBM 4 (+)	vIII	ND
HNSCC 5 (+)	ND	ND	GBM 5 (+)	vIII	ND
			GBM 6 (+)	vIII	ND

^a^(+/-) indicate EGFRvIII status of tumor sample by RT-PCR

^b^ND: EGFRvIII not detected

^c^vIII: EGFRvIII detected.

### Long-range PCR of intron 1 in HNSCC and GBM reveals genomic EGFRvIII without mRNA expression

The lack of genomic evidence for EGFRvIII in some assays may be due to large portions of intron1 remaining intact despite deletion of exons 2–7. This would limit the ability of the assay to detect EGFRvIII due to the size of the amplified product. To determine if breakpoints in intron 1 that included large portions of intron 1 were present, we performed long range PCR. Four glioblastomas with EGFR gene amplification identified by FISH were used in long-range PCR of intron 1 in total RNA as well as DNA (Table 2, S1 Fig.). We found that the unspliced RNA of all three of the EGFRvIII positive tumors had breakpoints in intron 1 that connected to intron7/exon 8. Two of these tumors harbored the same breakpoint that was close to exon 1. The third tumor harbored two separate populations of breakpoints, one close to the end of intron 1, and the other about halfway through intron 1. All 4 EGFR gene amplified tumors showed a DNA breakpoint in intron 1 that connected to intron7/exon 8 (including the EGFRvIII negative by mRNA). The tumor that did not have EGFRvIII mRNA had only one breakpoint. The other three tumors had multiple breakpoints, several of which overlapped with the unspliced RNA breakpoints.

**Table 2:** GBM long range PCR of EGFR intron 1.

Sample	vIII mRNA	EGFR amp	Pre mRNA	Full EGFR	Unique breakpts	Location^a^
171 RNA	+^b^	+^c^	+^d^	Yes	1	4676431
145 RNA	-	+	+	Yes	0	
159 RNA	+	+	+	Yes	1	4676431
111 RNA	+	+	+	Yes	2	4790760, 4723310/4724521
171 DNA	+	+	NA	NA	2	4790760, 4784752
145 DNA	-	+	NA	NA	1	4772858
159 DNA	+	+	NA	NA	2	4790760, 4751751
111 DNA	+	+	NA	NA	4	4786147, 4790760, 4712854, 4696307

^a^ The NCBI reference sequence for EGFR GRCh37.p10 was used with NT_033968.6 for DNA and NM_005228.3 for mRNA

^b^ mRNA with EGFRvIII transcript is denoted as “+”, wtEGFR only transcript is denoted as “-“

^c^ Tumor samples with EGFR gene amplification are denoted by “+”

^d^ Tumor samples with Exon 1 to 8 joining at the pre mRNA level are denoted as “+”.

Seven HNSCC tumors with and without EGFR gene amplification were also screened (Fig. 2c). The seven tumors were chosen based on fresh frozen tissue availability, and known EGFR gene amplification and EGFRvIII status (Table 3). Analysis of unspliced RNA showed that only one sample (EGFRvIII positive and EGFR gene amplification positive) demonstrated an EGFRvIII breakpoint. Genomic DNA analysis demonstrated that an EGFRvIII breakpoint was present for all but one sample (this sample was EGFRvIII positive but EGFR amplification negative). The greatest number of unique EGFRvIII breakpoints was found in EGFRvIII negative HNSCC samples without EGFR gene amplification.

**Table 3:** HNSCC long range PCR of EGFR intron 1.

Sample	vIII mRNA	EGFR amp	Pre mRNA	Full EGFR	Unique breakpoints	Location^a^
704 RNA	-^b^	+^c^	+^d^	Yes	0	0
061 RNA	-	+	-	Yes	0	0
035 RNA	+	+	-	Yes	0	0
836 RNA	+	+	+	Yes	1	4790760
021 RNA	+	-	-	Yes	0	0
681 RNA	-	-	-	Yes	0	0
120810 RNA	-	-	-	Yes	0	0
704 DNA	-	+	NA	NA	1	4676431
061 DNA	-	+	NA	NA	1	4676431
035 DNA	+	+	NA	NA	1	4676431
836 DNA	+	+	NA	NA	1	4676431
021 DNA	+	-	NA	NA	0	0
681 DNA	-	-	NA	NA	4	4784580, 4676431, 4790760, 4724521
120810 DNA	-	-	NA	NA	4	4784752, 4676431, 4790760, 4724521

^a^ The NCBI reference sequence for EGFR GRCh37.p10 was used with NT_033968.6 for DNA and NM_005228.3 for mRNA

^b^ mRNA with EGFRvIII transcript is denoted as “+”, wtEGFR only transcript is denoted as “-“

^c^ Tumor samples with EGFR gene amplification are denoted by “+”

^d^ Tumor samples with Exon 1 to 8 joining at the pre mRNA level are denoted as “+”.

### RNA sequencing analysis may not identify EGFRvIII in HNSCC tumors

We next interrogated EGFRvIII interrogation at the mRNA level to look at possible alternative splicing mechanisms. RNASeq was performed on 23 HNSCC RNA specimens (including the 7 used for long-range PCR) that had previously been screened by RT-PCR for EGFRvIII as well as on the U87MG cell line, which was previously engineered to exogenously express EGFRvIII [25]. No transcripts corresponding to EGFRvIII were identified in any of the samples by automated fusion detection. However, on manual review, reads corresponding to EGFRvIII were readily apparent in U87MGvIII cell line (Table 4). To probe this discrepancy in detail we assessed the reads corresponding to EGFRvIII in U87MG manually using Integrative Genomics Viewer. As expected from the automated fusion calling results, no reads were seen indicating a fusion of exons 1 and 8. However, an abnormal *EGFR* transcript was readily observed in which multiple reads aligning to exon 1 appeared to be fused to a region of intron 1 beginning at position chr7:55111526. Inspection of this intronic region revealed homology to exon 8 of *EGFR* with a four base mismatch in the first ten bases. All 38 reads in this configuration demonstrated alignment to this intronic region with a four base mismatch, suggesting misalignment of the reads to this intronic region instead of to exon 8 (Table 4). These results raise the possibility that some automated fusion detection algorithms may be limited in their ability to detect EGFRvIII without manual review or additional optimization efforts.

**Table 4:** Summary of RNA Sequencing Detection of EGFRvIII.

Sample	Exon 1 peak coverage	Exon 8 peak coverage	Reads supporting normal	Reads supporting vIII	Fusion reads aligning to intron 1
U87MGvIII	597	1199	67	38	0
131	4	1	0	0	0
2010	6	2	1	0	1
21	4	3	3	0	0
210	17	20	18	0	8
21b^a^	7	1	5	0	0
274	94	69	107	0	3
31111	26	15	30	0	0
35	20	4	7	0	1
35b^b^	31	15	21	0	0
396	19	12	18	0	1
442	69	34	43	0	2
443	10	11	14	0	0
477	34	34	56	0	1
561	20	18	15	0	2
61	15	18	13	0	0
615	16	22	26	0	1
669	58	66	86	0	1
681	4	5	2	0	0
704	32	19	30	0	1
723	9	6	7	0	0
725	64	54	82	0	1
80	3	2	4	0	0
810	6	8	10	0	0
836	4	2	3	0	0
836b^c^	2	2	0	0	0

^a^ Sample 21b is a separate RNA extraction from specimen 21.

^b^ Sample 35b is a separate RNA extraction from specimen 35.

^c^ Sample 836b is a separate RNA extraction from specimen 836.

Lower coverage of *EGFR* was observed in the 23 HNSCC tumors compared to the U87MG cell line (exon 1 peak coverage in the HNSCC tumors ranged from 2–94 reads as compared to 597 reads in the cell line). In 12 of the 23 HNSCC tumors, one to three transcripts were observed which demonstrated fusion of exon 1 of *EGFR* to the exact intronic region noted to likely represent EGFRvIII in U87MG. However, in contrast to U87MG cells, the reads perfectly aligned to the reference intronic sequence in spans ranging from 1–91 base pairs. This region, which follows a canonical splice acceptor site, is likely to be a component of an alternate *EGFR* transcript which is expressed at low levels. The homology of this region to exon 8 of *EGFR* could be problematic in designing 3’ oligos aimed at detecting *EGFR vIII* transcripts at the mRNA level, which underscored the need for the specifically designed primers and sequencing used in this work for RT-PCR detection.

## Discussion

EGFRvIII is a variant form of EGFR that contains an in-frame deletion of exons 2–7 of the external ligand-binding domain. This EGFR variant is constitutively active in the absence of ligand and does not appear to bind ligand [26]. EGFRvIII is tumor-specific and therefore represents an ideal cancer therapeutic target [27]. In HNSCC, EGFRvIII biology is incompletely understood and mechanism(s) of EGFRvIII expression have not been explored. Elucidation of the biology of EGFRvIII expression and signaling is needed to fully understand how to optimize the treatment of EGFRvIII expressing tumors. We undertook this study to address potential mechanisms responsible for EGFRvIII generation in HNSCC with the ultimate goal of overcoming cetuximab resistance.

Genomic deletion of exons 2–7 through EGFR gene amplification has been widely accepted as the primary, if not exclusive, mechanism of EGFRvIII expression in glioblastoma [10,28]. The restriction of EGFRvIII expression to EGFR amplified brain tumors supports this hypothesis [10,18]. However, we found that in HNSCC, EGFRvIII expression by RT-PCR with specifically designed primers and subsequent Sanger sequencing is equally common in both EGFR amplified and unamplified tumors (Fig. 1b); a finding supported by a previous report in a smaller cohort of HNSCC [19]. We also found that EGFRvIII does not appear to be common in p16/HPV-positive positive tumors, although few tumors were tested. This is in agreement with a previous study [21] and suggests that EGFRvIII tumors may be more common in HPV-negative HNSCC, which are generally considered to be associated with a worse prognosis compared with HPV-positive disease (Fig. 1c).

Alternative splicing due to point mutations or RNA editing does not appear to be a mechanism of EGFRvIII expression in HNSCC or glioblastoma, as we found no alterations in splice acceptor or donor sites in genomic DNA and unspliced RNA. There are many known and hypothesized mechanisms of regulating alternative splicing and alternative splicing, in general, is found to be less common in cancer than in normal tissue [29]. There have been reports, however, that alternative splicing can create oncogenes that drive cell motility in breast and colon cancers [30]. Our results indicate that alternative splicing may contribute to EGFRvIII expression in HNSCC, but is not the only mechanism that generates EGFRvIII in HNSCC tumors.

Our long range PCR analysis of HNSCC and glioblastoma samples revealed a large number of breakpoints in intron 1 of EGFR that resulted in loss of exons 2–7 at the genomic level. Intron 1 of many genes, including EGFR, are known to have important regulatory functions for transcription and translation [31]. The first intron of EGFR harbors a downstream enhancer element in close proximity to a polymorphic simple sequence repeat with 14–21 CA dinucleotides where the most frequent allele contains 16 CA repeats [32]. The presence of allelic imbalance in CA repeats may indicate increased genomic instability in this area, which may also contribute to a fragile site predisposed for chromosomal strand break [33]. Additionally, intron 1 contains 11 short interspersed Alu elements that are approximately 300 nucleotides in length and have been shown to be involved in non-allelic homologous recombination in several types of cancers [34]. A previous study of EGFRvIII in glioblastoma showed that Alu elements may be involved in intragenic rearrangement of EGFR to express EGFRvIII [22].

Our long-range PCR analysis suggested that loss of exons 2–7 at the genomic level does not always lead to mRNA transcript expression of EGFRvIII in HNSCC or glioblastoma. This could be due to heterogeneity in the tumor sample with few cells containing DNA alterations and the subsequent mRNA transcripts being below the detectable limits of our assays. In glioblastoma, EGFRvIII protein expression is known to be heterogeneous and unless a DNA, RNA and protein analysis can be performed on the same part of the tumor there is a potential for inconsistency of EGFRvIII detection results. A recent study in glioblastoma cell lines indicated that epigenetic mechanisms may also contribute to EGFRvIII expression in the presence of EGFRvIII genomic alterations [35].

RNAseq is a commonly used high-throughput methodology for measuring mRNA in tissue specimens and was recently employed to evaluate EGFRvIII in glioblastoma specimens from the The Cancer Genome Atlas (TCGA) [36]. In this retrospective manual analysis, we found that EGFRvIII mRNA was easily missed using this approach and required manual alignment to view putative EGFRvIII. In the HNSCC TCGA cohort, the presence of EGFRvIII was not supported by the RNAseq data. However, this analysis is likely limited by low coverage of *EGFR* in some tumors and further complicated by homology among a specific region of intron 1 of *EGFR* and the beginning of exon 8. In light of these findings, it is therefore possible that the paucity of EGFRvIII detected in large scale analyses utilizing automated sequence alignment tools (such as the TCGA) cannot be considered definitive proof of the absence of EGFRvIII. Further, the use of RT-PCR methods may be susceptible to false positives by identifying alternate short EGFR transcripts which are not EGFRvIII. It is likely that highly multiplexed PCR-based detection with carefully designed oligonucleotide pairs followed by deep sequencing would improve both the sensitivity and specificity of EGFRvIII detection as compared to conventional RT-PCR and RNAseq.

EGFR tyrosine kinase inhibitors (TKIs), which interfere with EGFR signal transduction, have also shown activity against EGFRvIII in preclinical models and in some clinical trials [37]. The EGFR TKI erlotinib, in particular, has been extensively tested in EGFRvIII expressing glioblastoma, but the results are inconsistent [38,39]. In HNSCC, EGFRvIII expression was associated with better disease control overall in a retrospective study, but there was no selective advantage to treatment with erlotinib [40]. A phase II clinical trial in HNSCC is currently assessing the efficacy of a pan-HER inhibitor in a cohort that will be evaluated for EGFRvIII expression [41]. Despite reduced phosphorylation of EGFR and EGFRvIII with TKI treatment downstream signaling pathways remain activated likely due to crosstalk and feedback of intracellular signaling in cancer cells [42]. The anti-EGFR monoclonal antibody cetuximab is less likely to bind the altered extracellular domain of EGFRvIII, which may mitigate clinical responses in HNSCC [43].

Our results suggest that while 10–40% of HNSCC express EGFRvIII by IHC or PCR methodologies, the level of expression of EGFRvIII in these tumors is low. Additionally, intratumoral heterogeneity of EGFRvIII expression may result in a subpopulation of cells that express this EGFR variant, whose contribution to EGFR targeted therapies remains incompletely defined. Our results highlight the difficulty in studying this low expression protein and suggest that no single mechanism results in EGFRvIII in human cancers. It appears that in HNSCC, EGFRvIII can arise independently of EGFR gene amplification and, moreover, that alteration at the gene level does not always lead to mRNA expression of EGFRvIII transcript. The use of multiple methodologies here underscores the difficulties in accurately detecting EGFRvIII and emphasizes the need for carefully designed assays. EGFRvIII represents an attractive therapeutic target as well as a potential negative predictive biomarker for cetuximab therapy in HNSCC. Improved detection methods will allow the design of trials, which assess the contribution of this altered form of EGFR to treatment responses.

## Supporting Information

S1 Fig Schematic of Long-Range PCR Results. Schematic representation of data in tables 2 and 3.
(PPTX)

S1 Methods PCR Methods Details. (DOCX)

S1 Table Long-Range PCR Primer List. (XLS)
