# Supplementary material for: Challenges in EGFRvIII Detection in Head and Neck Squamous Cell Carcinoma
Source: PLoS One. 2015 Feb 6;10(2):e0117781. doi: 10.1371/journal.pone.0117781 (PMC4320077; doi:10.1371/journal.pone.0117781)
Supplement: S1 Fig — Schematic representation of data in tables 2 and 3. (PPTX) [file pone.0117781.s001.pptx]

## Slide 1
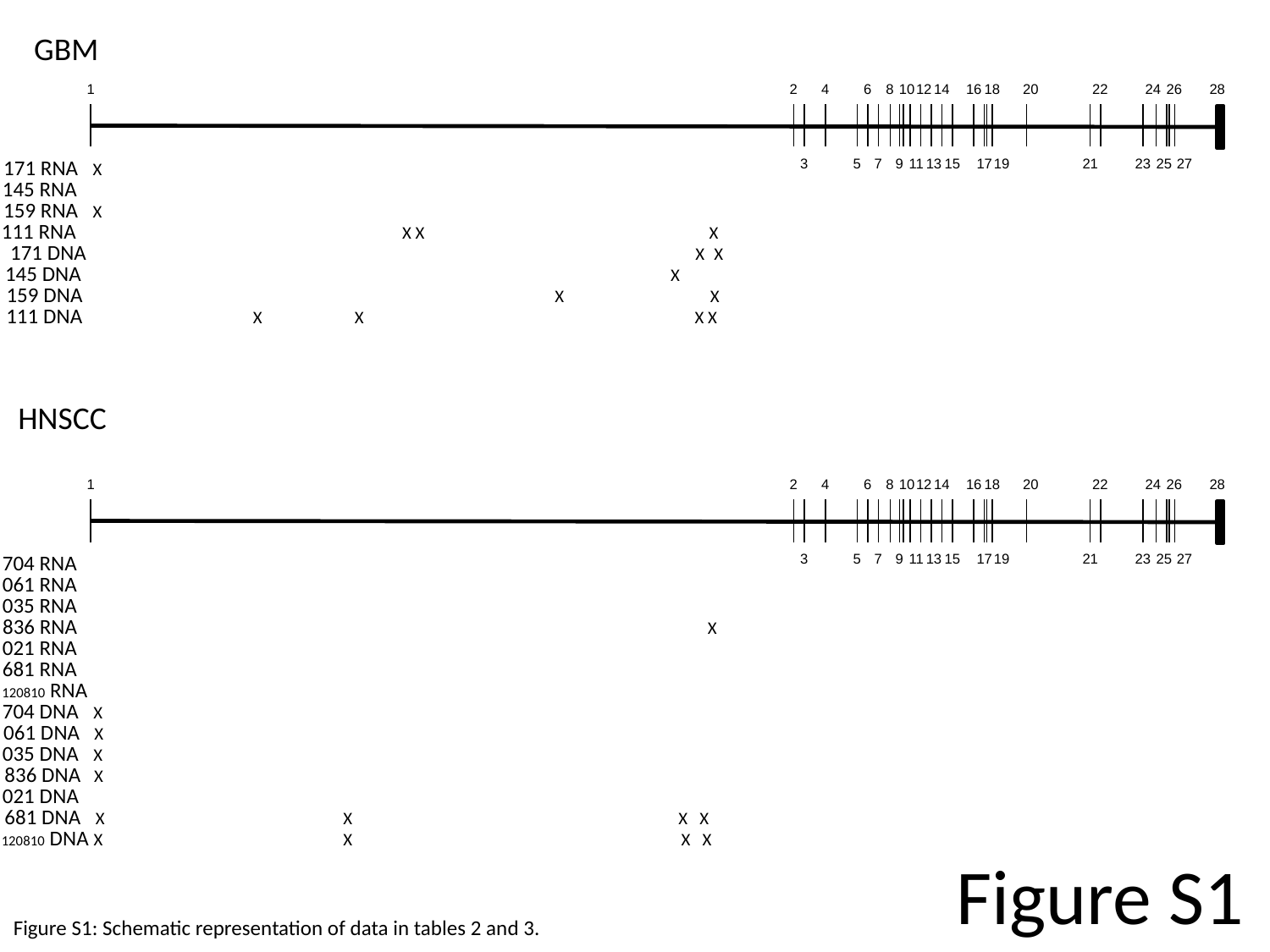

GBM
1
2
4
6
8
10
12
14
16
18
20
22
24
26
28
171 RNA X
3
5
7
9
11
13
15
17
19
21
23
25
27
145 RNA
159 RNA X
111 RNA X X X
171 DNA X X
145 DNA X
159 DNA X X
111 DNA X X X X
HNSCC
1
2
4
6
8
10
12
14
16
18
20
22
24
26
28
704 RNA
3
5
7
9
11
13
15
17
19
21
23
25
27
061 RNA
035 RNA
836 RNA X
021 RNA
681 RNA
120810 RNA
704 DNA X
061 DNA X
035 DNA X
836 DNA X
021 DNA
681 DNA X X X X
120810 DNA X X X X
# Figure S1
Figure S1: Schematic representation of data in tables 2 and 3.
