# Supplementary material for: Challenges in EGFRvIII Detection in Head and Neck Squamous Cell Carcinoma
Source: PLoS One. 2015 Feb 6;10(2):e0117781. doi: 10.1371/journal.pone.0117781 (PMC4320077; doi:10.1371/journal.pone.0117781)
Supplement: S1 Methods — (DOCX) [file pone.0117781.s002.docx]

**Supplementary methods S1**

EXON JUNCTION SEQUENCING

Primers designed to flank the end of EGFR exon 1 and the following intron

Fwd 5’ CCAGTATTGATCGGGAGAGC 3’; Rev 5’ CGCAGCTGATCTCAAGGAAA 3’

Primers designed to flank the beginning of exon 2 and the previous intron

Fwd 5’ TGGACCTTGAGGGATTGTTT 3’; Rev 5’ CTTCAAGTGGAATTCTGCCC 3’

Primers designed to flank the end of exon 7 and the following intron

Fwd 5’ GCTTTCTGACGGGAGTCAAC 3’; Rev 5’ AGACAGAGCGGGACAAGGAT 3’

Primers designed to flank the beginning of exon 8 and the previous intron

Fwd 5’ CTTTCCATCACCCCTCAAGA 3’; Rev 5’ CTCAGCAGCCGAGAACAAG 3’

Primers located in exon 1 and in the intron following exon 8 for detection of EGFRvIII at the genomic and unspliced mRNA levels

Fwd 5’ CAGTATTGATCGGGAGAGCC 3’; Rev 5’ CACAACCTTCAGTGCCTTCC 3’

GAPDH Primers

Fwd 5’ TGGAATTTGCCATGGGTG 3’; Rev 5’ GTGAAGGTCGGAGTCAAC 3’

PCR was performed using the Phusion High Fidelity PCR master mix (New England BioLabs, Inc.; Ipswich, MA) with 500 ng DNA or 375 ng cDNA. PCR was performed with an initial start of 98°C for 30 seconds. Denaturation, annealing, and extension were performed at 98°C (10 seconds), 56°C (30 seconds) (60°C for exon1 primer set), and 72°C (30 seconds), respectively, for a total of 38 cycles. The reaction was completed with an extension period at 72°C for 10 minutes.

LONG RANGE PCR

PCR was performed using Advantage GC Genomic LA Polymerase Mix (Clontech, Mountain View, CA). PCR reactions were run according to manufacturer’s protocol with 125ng cDNA or 100ng DNA. Reactions were initiated with 94°C for 1 minute, denaturation, annealing, and extension were done at 94°C (30 seconds), 60°C (30 seconds), and 72°C (4 minutes), respectively, for a total of 35 cycles. The reaction was completed with an extension period at 72°C for 5 minutes.
